# Genome-Wide Screening for Novel Candidate Virulence Related Response Regulator Genes in *Xanthomonas oryzae* pv. *oryzicola*

**DOI:** 10.3389/fmicb.2018.01789

**Published:** 2018-08-07

**Authors:** Zheng-Chun Zhang, Min Zhao, Li-Dan Xu, Xiang-Na Niu, Hong-Ping Qin, Yi-Ming Li, Mei-Lin Li, Zhong-Wei Jiang, Xia Yang, Guang-Hui Huang, Wei Jiang, Ji-Liang Tang, Yong-Qiang He

**Affiliations:** ^1^State Key Laboratory for Conservation and Utilization of Subtropical Agro-bioresources, College of Life Science and Technology, Guangxi University, Nanning, China; ^2^College of Agronomy, Guangxi University, Nanning, China

**Keywords:** *Xanthomonas oryzae* pv. *oryzicola*, two-component regulatory system, response regulator, genome-wide mutagenesis, adaptation, virulence

## Abstract

Two-component regulatory system (TCS), a major type of cellular signal transduction system, is widely used by bacteria to adapt to different conditions and to colonize certain ecological niches in response to environmental stimuli. TCSs are of distinct functional diversity, genetic diversity, and species specificity (pathovar specificity, even strain specificity) across bacterial groups. Although TCSs have been demonstrated to be crucial to the virulence of *Xanthomonas*, only a few researches have been reported about the studies of TCSs in *Xanthomonas oryzae* pathovar *oryzicola* (hereafter *Xoc*), the pathogen of rice bacterial streak disease. In the genome of *Xoc* strain GX01, it has been annotated 110 TCSs genes encoding 54 response regulators (RRs), 36 orthodox histidine kinase (HKs) and 20 hybrid histidine kinase (HyHKs). To evaluate the involvement of TCSs in the stress adaptation and virulence of *Xoc*, we mutated 50 annotated RR genes in *Xoc* GX01 by homologous vector integration mutagenesis and assessed their phenotypes in given conditions and tested their virulence on host rice. 17 RR genes were identified to be likely involved in virulence of *Xoc*, of which 10 RR genes are novel virulence genes in *Xanthomonas*, including three novel virulence genes for bacteria. Of the novel candidate virulence genes, some of which may be involved in the general stress adaptation, exopolysaccharide production, extracellular protease secretion and swarming motility of *Xoc*. Our results will facilitate further studies on revealing the biological functions of TCS genes in this phytopathogenic bacterium.

## Introduction

Two-component regulatory system (TCSs), also known as two-component signal transduction systems, are widely used by bacteria to adapt to different conditions and to colonize specific ecological niches in response to environmental signals (Stock et al., 2000). A typical TCS consists of a sensor with membrane-bound histidine kinase (HK) that senses a specific environmental clues and an associated response regulator (RR) with certain output domains that mediate the differential expression of target genes or the cellular level of the signal molecules, through a phosphorelay from sensor to regulator (Stock et al., 2000). TCSs regulate many bacterial characteristics such as adaptation, metabolism, motility, pathogenicity and virulence, which are regarded as one of the important indicators for measuring the bacterial adaptive potential, i.e., the so-called bacterial intelligence quotient (bacterial “IQ”) (Galperin, 2005). The absence of TCS proteins in most eukaryotic genomes makes them the ideal potential targets for novel antimicrobial drug design (Barrett et al., 1998). Thus, TCSs attracts great attentions from both in medicine and agriculture researches.

The genus *Xanthomonas*, belonging to the γ-Proteobacteria, is a diverse and economically important group of bacterial phytopathogens. *Xanthomonas* spp. can infect at least 124 monocotyledonous and 268 dicotyledonous plant species (Hayward, 1993). Different plants have different growth environments, internal niches and resistance mechanism (Zeng et al., 2010). To infect plants successfully, *Xanthomonas* pathogens have developed different adaptation and pathogenic mechanisms. Genomic surveys showed that *Xanthomonas* encodes approximately 110 TCS gene in average (Qian et al., 2008a). About 70 TCS genes are shared by all studied genomes of *Xanthomonas*, constituting a “core set” of TCSs which were generally regarded to carry out similar biological functions across *Xanthomonas* spp. (Wang et al., 2010). For example, the RpfC-RpfG system, the first and best-documented TCS identified in *Xanthomonas*, regulates the biosynthesis of several virulence factors during quorum-sensing, a cell-cell communication phenomenon whereby a single bacterial cell can sense and respond to the population density through detection of the concentration of signaling molecules (Tang et al., 1991; Zhang et al., 2013). HrpG, an OmpR-family RR, is the core regulator which controls the expression of the type III secretion system, the critical secretion machinery that translocates type III effectors (T3Es) into plant cells interfering with cellular processes to promote pathogen proliferation (Wengelnik et al., 1996). ColS-ColR (also named as VgrS-VgrR) is identified in *Xcc* by two independent studies (Qian et al., 2008b; Zhang et al., 2008). VemR, a standalone REC domain protein, positively regulates the virulence and adaptation of *Xcc*, which may regulate downstream factors via protein–protein interactions (Tao and He, 2010). Most recently, Wang et al. (2017) reported that PcrK, a HyHK type receptor from *Xcc* strain 8004, can specifically sense the plant cytokinin and regulate its autokinase activity, and PcrK-PcrR regulates oxidative stress response and bacterial virulence under cytokinin stimulation. The findings might reveal a mechanism by which phytopathogens intercept a plant hormone to protect themselves from plant innate immunity (Wang et al., 2017). However, there are still considerable differences in the functions and signaling mechanisms of orthologous TCSs in different xanthomonads. For example, the RavS-RavR system is another TCS that is associated with c-di-GMP turnover. It was reported that mutation in *ravR* gene did not result in virulence deficiency of *Xcc* 8004 (Osbourn et al., 1990). But, knockout of *ravR* orthologs in *Xcc* ATCC 33913 and *Xcc* XC1 led to attenuation in virulence, suggesting that strain-specificity exists in the regulatory function of some TCSs (Qian et al., 2008b; He et al., 2009). In *Xcc* strain 8004, HrpG can partially be activated by a sensor kinase HpaS through phosphorylation (Li R.F et al., 2014). HpaS is a HK/RR hybrid protein. Its coding gene, *hpaS*, resides in a highly diversified operon across *Xanthomonas* genomes. Even the closely related strains of *Xcc*, 8004 and B100 encode two HpaS proteins with the different sizes (Vorhölter et al., 2008). In *Xcc* 33913, mutation in *hrpG* only has limited effects on the virulence (Qian et al., 2008b), which differs from mutational analyses of its orthologs in *Xcc* 8004 (Qian et al., 2005) and *Xcv* 85-10 (Wengelnik et al., 1996). Additionally, the non-core TCSs are expected to play the certain role in adapting specific niches and infecting certain hosts. For example, the mutant of *XCC1187* in *Xcc* ATCC33913 was found significantly reduced survival under many stress conditions tested (Qian et al., 2008b), but its homologs are absent both in *Xoc* and *Xoo*. The above studies suggest that the bacterial TCSs are more complex than anticipated.

In recent years advances have been made in the understanding of the biological roles and basic mechanisms of signaling in bacterial TCSs. A genome-scale mutagenesis and phenotypic assays of TCSs were studied in *Xanthomonas campestris* pv. *campestris* ATCC 33913, four RR genes had been demonstrated in the full virulence for *Xcc* (Qian et al., 2008b). To date, 20 TCS proteins have been identified to be related to pathogenesis of *Xanthomonas*, including eight pairs of typical TCSs, RpfC-RpfG (Tang et al., 1991), RaxH-RaxR (Burdman et al., 2004), PhoQ-PhoP (Lee et al., 2008), ColS-ColR (also named VgrS-VgrR) (Qian et al., 2008b; Zhang et al., 2008; Noh et al., 2014; Wang et al., 2016), RavS-RavR (He et al., 2009), PdeK-PdeR (also named RavA-RavR) (Yang et al., 2012; Tao et al., 2014), HpaR2-HpaS (Li R.F et al., 2014), PcrK-PcrR (Wang et al., 2017), four orphan RRs HrpG (Wengelnik et al., 1996), VemR (Tao and He, 2010), BfdR (Huang et al., 2013), GsmR (Liu et al., 2013), and XerR (also named XmbR and XibR) (Wang et al., 2011; Yaryura et al., 2015; Pandey et al., 2016). The TCSs modulate the expression of several virulence factors through diverse molecular mechanisms such as interacting with DNA, protein-binding and involvement in second messenger metabolism, which generates a high level of regulatory versatility (Qian et al., 2008a; Wang et al., 2010).

*Xanthomonas oryzae* pv. *oryzicola* (*Xoc*) is the causal agent of rice bacterial leaf streak (BLS), one of the major bacterial diseases of rice with approximately 10–30% yield loss in epidemic regions (Niño-Liu et al., 2006). The pathogen infects rice leaf mainly through stomata and wounds and colonizes apoplast of the mesophyll cells, resulting in interveinal necrotic lesions, from which the disease name designated (Fang et al., 1957). Genomic surveys indicated that the genome of *Xoc* strain BLS256 consists of approximately 107 putative genes encoding TCSs, some of which are absent from the genomes of *Xcc* and other *Xanthomonas* spp. (Bogdanove et al., 2011). Recently, we have sequenced the complete genome of *Xoc* strain GX01, a Chinese *Xoc* isolate (Zhao et al., 2012; Niu et al., 2015). The genome annotation revealed that the two *Xoc* strains, BLS256 and GX01, are conserved in TCS genes but three more HK genes in GX01, although the latter harbors an indigenous plasmid (Niu et al., 2015). Comparative genome analysis also showed that TCS genes are significantly difference between *Xanthomonas* species, even between the pathovars in the same species. With the goal of elucidating the role of TCS system in the pathogenesis of *Xoc*, in this study, we used a reverse-genetic approach to mutate all putative RR genes in *Xoc* GX01 by integration mutagenesis method, and characterized the phenotype of mutants by examining multiple stress tolerances, virulence, and some virulence determinants. Our results presented a comprehensive insight into the contribution of RR genes in regulation of responses to diverse environmental conditions as well as virulence of *Xoc*.

## Materials and Methods

### Bacterial Strains, Plasmids and Growth Conditions

The related bacterial strains and plasmids used in this study are listed in **Table 1**. *X. oryzae* pv. *oryzicola* GX01 are usually cultured under 28°C in nutrient medium NB (3.0 g/L beef extract; 5.0 g/L polypeptone; 1.0 g/L yeast extract; 10.0g/L sucrose; pH7.0), nutrient agar medium NA (NB plus 8.0 g/L agar) (Siciliano et al., 2006) and minimal medium MMX (2.0 g/L [NH_4_]_2_SO_4_, 4.0 g/L K_2_HPO_4_, 6.0 g/L KH_2_PO_4_, 0.2 g/L MgSO_4_⋅7H_2_O, 1.0 g/L citric acid, 5.0 g glucose) (Daniels et al., 1984). *Escherichia coli* strains were cultured in LB medium (Sambrook et al., 1989) at 37°C. The competent cells of *E. coli* were cultured under 37°C in liquid SOB medium (20 g/L peptone; 5 g/L yeast extract; 0.5 g/L NaCl; 2.5 mM KCl, pH7.0). If required, appropriate concentrations of antibiotics can be added to the culture medium, for example: ampicillin (Amp), 100 μg/mL; kanamycin (Kan), 25 μg/mL; rifampicin (Rif), 50 μg/mL; tetracycline (Tc), 15 μg/mL for *E. coli*, 5 μg/mL for *Xoc*.

**Table 1:** Bacterial strains and plasmids used in this study.

Strain or plasmid	Genotype or description	Reference
Plasmids		
pK18mob	Suicide vector to create a mutant by a single crossover; Kan^r^	Schäfer et al., 1994
pK18mobsacB	Suicide vector to create mutant by double crossover recombination; Kan^r^	Schäfer et al., 1994
pLAFRJ	Shuttle plasmid pLAFR3 derivate containing the multiple cloning sites of pUC19; Tc^r^	Jiang et al., 2009
Strain		
*Xoc* GX01	Wild-type strain; Rif^r^	Lab collection
*E. coli* DH5α	Used for molecular cloning	Lab collection
NK0263–NK4109 (a total of 50 strains)	*GX_XOC0263* to *GX_XOC4109* (a total of 50 genes), mutants with insertions of response regulator genes of *Xoc* GX01, constructed by pK18mob vector integration; Kan^r^	This study
CNK2201	Genetic complementary strain of NK2201 (mutant of *GX_XOC2201*); Rif^r^, Kan^r^, Tc^r^	This study

### Construction of Insertion Inactivation Mutants and Genetic Complementarity

To generate insertional mutants of RR genes, a relatively conservative gene fragment within the functional domain of the target gene was amplified by PCR, using the total DNA of the wild-type strain as the template, and cloned into the suicide plasmid pK18mob in the same expression orientation of Plac promoter upstream of multiple cloning site (MCS) in pK18mob (Schäfer et al., 1994). After confirmation by PCR and sequencing, the constructed plasmid was transferred into the wild-type strain GX01 by triparental conjugation. Kan-resistant transconjugants were selected and further confirmed by PCR. Three of confirmed transconjugants for each RR gene were randomly chosen and stored, one of which designated as RR mutants for further study.

For functional restoration of the RR mutants, a DNA fragment that includes the intact protein coding DNA sequence (CDS) region of each RR gene was amplified by PCR using the total genome DNA of strain GX01 as template. After confirmation by sequencing, the amplified DNA fragment was cloned into pLAFRJ (Jiang et al., 2009) to generate the recombinant plasmid. The recombinant plasmid was then transferred into the RR mutant by triparental conjugation. The transconjugants were screened on NA supplemented with Rif, Kan and Tc. Three of confirmed transconjugants were randomly chosen and stored, one of which was representatively chosen for further study.

### Plate Assays of Phenotypic Characterization

*Xoc* strains to be tested were grown in NB medium overnight, and the suspensions were spectrophotometrically adjusted to OD_600_ = 0.1. The phenotypes of each RR mutants were assessed by using plate assays, the rapid and semi-quantitative methods for bacterial EPS production and extracellular enzyme activity, which were judged by the colonial morphology and size or the size of the hydrolysis transparent ring around the colony, respectively.

For the EPS assays, 2 μL of the cell suspension (OD_600_ = 0.1) of each strain was pipetted onto NA with 2% sucrose. A five-degree scoring system had been established for EPS plate assay, adapted from the method by Tang et al. (1991), in which EPS production of each strain was scored by the colonial morphology and size. EPS production of the wild type strain *Xoc* GX01 is deliberately set as degree III. Degree I is for the least EPS production, and Degree V is for the most EPS production. Degree II is the median between Degree I and III, and Degree IV is the median between Degree III and V.

For the extracellular protease activity assays, 2 μL of the cell suspension (OD_600_ = 0.1) of each strain was pipetted onto NA with 1% skim milk. The difference of extracellular protease could be judged by comparing the availability or size of the hydrolysis transparent ring around the colony, and the results were the average of three independent experiment (Tang et al., 1991).

For the swarming assays, 2 μL of the cell suspension (OD_600_ = 0.1) of each strain was pipetted onto the semi-solid NB medium with 0.6% agar and cultured for 3 days at 28°C. The diameters of the swarming zones were measured, and the results were the average of three independent experiment (Zheng et al., 2016).

### Stress Tolerance Assays

Experiments were made to determine the relative survival rate of bacteria under four environmental stresses, including high osmotic pressure, heavy metal stress, antioxidant reactions, and membrane disruption (Zhang et al., 2008; Niu et al., 2015). The *Xoc* strains were cultured overnight in NB Medium, were diluted to the concentration of OD_600_ = 1.0, and then the 10% fresh bacterial suspensions was inoculated to NB, respectively, which contain 300 mM NaCl, 12 μM CdSO_4_, 0.125 mM H_2_O_2_, 8 × 10^-5^ g/ml sodium dodecyl sulfate (SDS), the bacterial survival rate in the culture was measured after 16 h. The serial dilutions of each stress were plated on NA with certain antibiotics and incubated for 48 h. The bacterial colonies were counted to check the survival ability. The survival rate of each strain was the ratio of the bacterial survival in stress test to the normal condition, and the relative survival rate of certain mutant was the proportion of mutant survival rate to that of the wild type survival rate. Each stress test, plated in duplicate, was repeated at least three times. A Student’s *T*-test was used to evaluate the significance of the differences.

### Virulence Assays

In virulence assays, the bacterial suspensions concentration of each *Xoc* strain was adjusted to OD_600_ = 0.5 by the sterilized deionized water. Bacterial suspensions were infiltrated into the rice leaves (6 weeks), on the back of the main vein side, by using needleless syringe (Wang et al., 2007). Wild-type strain and sterilized deionized water were used as controls. Each strain was infiltrated 20 points. The plants were grown in a greenhouse at approximately 28°C, with a relative humidity > 90%. Lesion length was measured 14 days after inoculation and virulence level was scored in percentage of the lesion lengths inoculated by mutant and wild type strain.

### Hypersensitive Response Assays

The ability of the bacterial strains to elicit a hypersensitive response (HR) in an intact tobacco (*Nicotiana benthamiana*) leaf was tested by using infiltration method (Reimers and Leach, 1991). The RR mutant and wild strain were cultured to the logarithmic growth phase in NB medium containing the proper antibiotics. The bacterial suspensions were diluted in 10 mM MgCl_2_ at an approximate OD_600_ of 0.5 and spot-infiltrated in tobacco leaves by using a needleless syringe. The formation of water soaking spot on the leaves were observed at 48 and 72 h after infiltration. All infiltrations were repeated at least three times.

### Growth Curve Determination

*Xoc* RR mutant strains were cultured in NB for about 18 h to the middle logarithmic phase. *Xoc* cells were then harvested, and finally adjusted to OD_600_ = 1.0. The resuspended *Xoc* culture was inoculated by 1%. The absorbance at OD_600_ was measured at certain times, and the values are expressed as mean ± standard deviation. Each *Xoc* strain was performed for three biological repeats.

## Results

### Genetic Diversity of TCS Genes in *Xanthomonas*

It was reported that xanthomonads possess a large repertoire of TCS genes which comprise approximately 3% of the putative CDSs of their genomes (Qian et al., 2008a; Wang et al., 2010). *Xcv* 85-10 encodes 126 TCS genes, the most TCS genes in one *Xanthomonas*, while as *X. albilineans* GPE PC73 has only 83 such genes, the least one with TCS genes^1^. A detailed genome surveys showed that *Xanthomonas* spp. are of significant genetic diversities in TCS genes (**Supplementary Table S2**), including the strain-specific genes that might be from gene gain, loss or duplication, and the slight allelic variations that might be generated from gene fusion, fission or point mutations. Each *Xanthomonas* species has 10 to 30 specific TCS genes (**Supplementary Table S1**). For example, compared with *Xcc* ATCC33913, *Xoc* GX01 has 18 strain-specific TCS genes, including *virG-virA* and *baeS-baeR* putative TCS pairs, and *fimX*, *nasT* and *qseB* genes. While *Xcc* ATCC33913 has 20 strain-specific TCS genes to *Xoc* GX01, including *bfdR-bsdS* and *desR-desK* pairs, and the unclassified ones (**Supplementary Table S1**). Interestingly, mutation in *XCC1187* (*bfdR*) resulted in significantly reduced survival under multi-stress conditions tested (Qian et al., 2008b). BfdR had been also demonstrated to link *rpf* with biofilm formation and virulence of *X. axonopodis* pv. *citri* (Huang et al., 2013).

Generally, horizontal gene transfer is the major contribution to strain-specific gene gain or deletion, however, genome decaying and gene fusion also led to distinct genetic diversity of TCS genes in *Xanthomonas*. For example, *hpaS* (*XC_3670*) was demonstrated to play very important role in HrpG phosphorylation in *Xcc* 8004 (Li R.F et al., 2014). However, its allelic genes are found to be absent from the genomes of some *Xanthomonas* spp., including all the sequenced *Xoo* strains. Sequence alignment showed that a short remnant sequence of *hpaS* remained in the allelic locus of the *Xoo* genomes, suggesting that a genome decaying collapsed *hpaS* in *Xoo*. In *Xac* 306, the *hpaS* allele encodes a large HK-RR hybrid protein, named StyS, which covers the HpaS and HpaR2 (XC_3669) in total length (da Silva et al., 2002). Comparative analysis showed that all the sequenced *X. citri* strains are almost same in this gene loci. This genetic rearrangement might be an early gene fusion event while the *X. citri* evolved divergently from *Xanthomonas* common ancestor. It is also worth noting that TCS pseudogenes contribute to the genetic diversity of TCS genes in *Xanthomonas*. Some examples are the genetic variations in *rpfC* alleles and *pcrK* alleles. RpfC-RpfG system is widespread in *Xanthomonadaceae*, playing a crucial role in cell-to-cell signaling. In a strain of *Xsa* strain R1, *rpfC* allele (*SB85_01470*) had been annotated as a pseudogene, owing to a single nucleotide deletion at position 1385th of the CDS resulted in a frameshift and an early stop subsequently. The predicted protein has no REC and HPT domains, which would lose biological function. For another example, the *pcrK* allele (*SB85_04760*) in *Xsa* strain R1 was also annotated as a pseudogene. In *Xcc*, PcrK was demonstrated to sense the cytokinin to enhance oxidative stress adaptation, which is probably the important bacterial feature to recognize the plants (Wang et al., 2017). Genetic diversity may lead to functional differences. Therefore, systematically functional characterization of TCS genes in a certain *Xanthomonas* spp. will not only dissect their biological roles in signaling transductions, but also facilitate to reveal the process of adaptive evolution in species differentiation of *Xanthomonas*. The detailed comparison of TCS genes between *Xoc* GX01 and some other important *Xanthomonas* spp. were list in **Supplementary Table S1**.

### Systematic Mutagenesis of RR Genes and Phenotypic Assays of Mutants in *Xoc* GX01

To generate a mutant library of RR genes in *Xoc* strain GX01, the internal DNA sequences (300–500 bp) of 54 RR genes (**Supplementary Table S1**) were amplified by PCR using the corresponding specific primers (**Supplementary Table S2**) and cloned into the suicide vector pK18mob to construct the recombinant plasmids (Schäfer et al., 1994). All 54 RR genes had been tried to mutate by insertional inactivation through single crossover using suicide recombinant plasmids. A total of 50 mutants were obtained and verified by PCR (**Table 1**). Four genes (*GX_XOC2472*, *GX_XOC3129*, *GX_XOC3720*, and *GX_XOC4069*) were unable to mutate, despite of several attempts. These genes might be essential for *Xoc* GX01 or the mutations might lead to serious defects in bacterial growth under the curtain condition or other uncertain factors in this study. Of the unmutated RR genes, *GX_XOC3720* has no ortholog in *Xcc* ATCC33913. Interestingly, of the immutable RR genes in *Xcc* ATCC33913, *XCC2695* has also no ortholog in *Xoc* GX01. The mutant of *XCC2180* in *Xcc*, the ortholog of *GX_XOC2472*, had significantly reduced survival rates under three stress conditions (Qian et al., 2008b). Coincidentally, *GX_XOC4069* and *XCC3943*, the mutual orthologs, could not be mutated in *Xoc* GX01 and *Xcc* ATCC33913 (Qian et al., 2008b). However, their ortholog in *Xoo* PXO99^Az^, encoding PhoP, could be mutated and had been demonstrated to be involved in pathogenicity of *Xoo* (Lee et al., 2008).

For estimating the effects of the mutagenesis of RR genes of *Xoc*, we have conducted a series of primary plate assays, including morphologic and auxotrophic assays. The results showed that all the RR mutants can grow on MMX agar medium, indicating that mutations in these RR genes did not lead to auxotroph. On the NA medium, each of the 50 RR mutants can develop into a yellow colony, suggesting that the interruption of RR genes did not affect the synthesis of xanthomonadin, the unique yellow pigment which is important for protection from photobiological damage (Poplawsky et al., 2000). On the NA plus 2% sucrose medium, the phenotype of the RR mutants varies significantly. The wild type *Xoc* strain produced a smooth, opaque, glistening, circular, convex and entire colony. The mutants of *GX_XOC2221*, *GX_XOC2338* (*virG*), and *GX_XOC3093* (*pilG*) generated big, glistening, circular, convex or mucoid colonies. The mutants of *GX*_*XOC0538*, *GX*_*XOC0882*, *GX*_*XOC1513* (*adeR*), *GX*_*XOC2201* (*rpfG*), *GX*_*XOC2201* (*vemR*), *GX*_*XOC2227* (*ravR*), and *GX*_*XOC3522* produced small, dry, flatten, crumpled or zigzag edged colonies. To confirm the authenticity of mutation effects, NK2201, the mutant of *GX_XOC2201* (*vemR*) was chosen as a representative for complementation assay. The result indicated that the plasmid pLARFJ carrying the intact *vemR* CDS region could fully restore the phenotypic alteration of the *GX_XOC2201* mutation. In addition, the mutants of three previously known EPS production associated RR genes (*rpfG, vemR and ravR*) were identified as small and dry colonies, together with complementation assay, verifying the effectiveness of our approaches.

### Impact of RR Mutation on *Xoc* EPS Synthesis, Extracellular Protease Activity and Swarming

Extracellular polysaccharides (EPS) and extracellular enzymes are virulence factors for many phytopathogenic bacteria (Tang et al., 1991). Swarming is also important for *Xanthomonas* invading hosts (Zheng et al., 2016). Investigations into EPS production, extracellular protease activity, and swarming ability of the RR mutants were performed by using plate assays.

#### EPS Production

**FIGURE 1:**
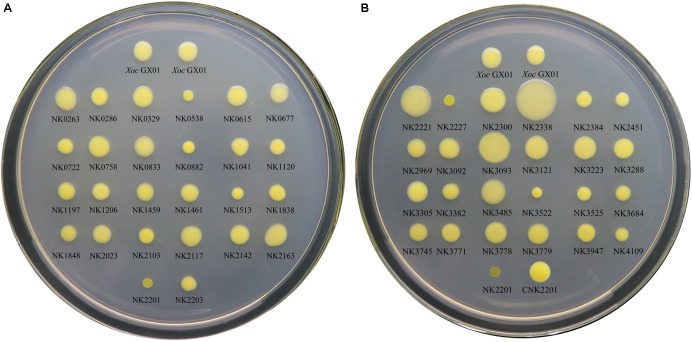
The EPS assays for all RR gene mutants of *Xoc* GX01 strain. The EPS biosynthesis ability of RR gene mutants was observed on solid medium NA plus 2% sucrose. The names of each mutant and the wild type strain were labeled beneath the colonies. One microliter of each bacterial culture (OD_600_ = 0.1) was inoculated on the plate, and the bacteria were grown for 72 h at 28°C. A five-degree scoring system had been established for EPS plate assay, adapted from the method by Tang et al. (1991), in which EPS production of each strain was scored by the colonial morphology and size. The degree of EPS production of each strain was listed in **Table 2**. **(A)** The EPS assays for RR gene mutant from *GX_XOC0263* to *GX_XOC2203*. **(B)** The EPS assays for RR gene mutant from *GX_XOC2221* to *GX_XOC4109* and genetic complementary strain of NK2201.

According the five-degree scoring system for determining EPS production, the results showed that six (NK0538, NK0882, NK1513, NK2201, NK2227, and NK3522) of the 50 RR gene mutants are classed as degree I, which means that they produced distinctly less amount of EPS in this study, and three mutants (NK2221, NK2338, and NK3093) are classed as degree V, indicating that they produced distinctly more amount of EPS (**Table 2** and **Figure 1**). Since the plate assay is only a semi-quantitative method, we only focus on the most distinctly altered mutants as the mutational effects, i.e., the mutants in degree I, performing dry, flatten, crumpled, zigzag edged and small sized colonies, and the mutants in degree V, showing glistening, circular, convex, mucoid and big colonies (**Table 2** and **Figure 1**). By checking the genes corresponding to these mutants, *rpfG, vemR and ravR* have been reported to play important roles in EPS biosynthesis (He et al., 2009; Tao and He, 2010). *adeR* (*GX_XOC1513*), and the homologs of *GX_XOC0538*, *GX_XOC0882*, and *GX_XOC3522* have no such reports, suggesting their novelties in positively regulating bacterial EPS production. It is also worth noting that the mutations in *GX_XOC2221*, *GX_XOC2338* (*virG*), and *GX_XOC3093* (*pilG*) substantially enhanced EPS production, which is uncommon in *Xanthomonas* spp.

**Table 2:** Plate assays of EPS production, extracellular protease activity and swarming ability of *Xoc* strains.

Gene ID	Gene name	Strain	EPS^a^	Swarming (%)^b^	Extracellular enzymes (%)^c^
		GX01	III	100	100
*GX_XOC0263*	*ntrC, glnG*	NK0263	III	76.19 ± 16.5	98.18 ± 14.55
*GX_XOC0286*	*ntrC family*	NK0286	III	88.1 ± 20.62	147.5 ± 2.82^*^
*GX_XOC0329*	*narL family*	NK0329	III	119.05 ± 16.5	87.08 ± 10.21
*GX_XOC0538*	*–*	NK0538	I	47.62 ± 16.5^*^	109.38 ± 8.06
*GX_XOC0615*	*algR*	NK0615	III	102.38 ± 20.62	87.4 ± 10.31
*GX_XOC0677*	*sreR*	NK0677	III	171.43 ± 12.37^*^	27.93 ± 14.28^*^
*GX_XOC0722*	*ompR family*	NK0722	III	88.1 ± 20.62	108.94 ± 2.77
*GX_XOC0758*	*kdpE*	NK0758	III	111.9 ± 16.5	114.1 ± 16.57
*GX_XOC0833*	*colR, detR*	NK0833	III	133.62 ± 16.5	93.41 ± 17.75
*GX_XOC0882*	*cheY family*	NK0882	I	40.48 ± 4.12^*^	107.01 ± 4.99
*GX_XOC1041*	*phoB*	NK1041	III	85.71 ± 12.37	52.77 ± 3.96^*^
*GX_XOC1120*	*pilH*	NK1120	II	90.48 ± 16.5	98.79 ± 11.45
*GX_XOC1197*	*colR, raxR*	NK1197	III	102.38 ± 20.62	87.06 ± 3.63
*GX_XOC1206*	*narL family*	NK1206	III	88.1 ± 20.62	80 ± 9.18
*GX_XOC1459*	*cheB1*	NK1459	III	95.24 ± 20.62	112.57 ± 10.18
*GX_XOC1461*	*vieA1*	NK1461	III	95.24 ± 20.62	108.7 ± 6.58
*GX_XOC1513*	*adeR*	NK1513	I	73.81 ± 8.25^*^	110.63 ± 5.32
*GX_XOC1838*	*pcrR*	NK1838	III	102.38 ± 20.62	109.78 ± 2.92
*GX_XOC1848*	*pleD*	NK1848	III	90.48 ± 16.5	88.1 ± 12.15
*GX_XOC2023*	*regA*	NK2023	III	126.19 ± 16.5	95.16 ± 8.36
*GX_XOC2103*	*rpfG*	NK2103	II	138.1 ± 20.62	52.01 ± 13.63^*^
*GX_XOC2117*	*cheB2*	NK2117	III	119.05 ± 16.5	106.39 ± 18.95
*GX_XOC2142*	*cheY*	NK2142	III	159.52 ± 20.62^*^	50.74 ± 0.95^*^
*GX_XOC2163*	*cheY*	NK2163	III	145.24 ± 20.62^*^	82.32 ± 21.29
*GX_XOC2201*	*vemR*	NK2201	I	59.52 ± 8.25^*^	96.05 ± 13.21
*GX_XOC2203*	*citB*	NK2203	II	128.57 ± 12.37	107.37 ± 7.86
*GX_XOC2221*	*cheV*	NK2221	V	166.67 ± 20.62^*^	52.45 ± 5.41^*^
*GX_XOC2227*	*ravR, pdeR*	NK2227	I	64.29 ± 12.37^*^	82.57 ± 17.56
*GX_XOC2300*	*–*	NK2300	IV	111.9 ± 16.5	79.66 ± 11.78
*GX_XOC2338*	*ompR, virG*	NK2338	V	280.95 ± 8.25^*^	72.82 ± 24.19
*GX_XOC2384*	*gacA*	NK2384	II	95.24 ± 20.62	80.23 ± 24.21
*GX_XOC2451*	*–*	NK2451	II	92.86 ± 12.37	51.04 ± 2.95^*^
*GX_XOC2969*	*–*	NK2969	III	95.24 ± 20.62	81.43 ± 19.36
*GX_XOC3092*	*pilH*	NK3092	III	109.52 ± 4.12	80.47 ± 16.24
*GX_XOC3093*	*pilG*	NK3093	V	245.24 ± 20.62^*^	88.76 ± 9.32
*GX_XOC3121*	*cheY family*	NK3121	IV	90.48 ± 16.5	98.86 ± 9.78
*GX_XOC3223*	*hrpG*	NK3223	IV	107.14 ± 12.37	93.75 ± 13.07
*GX_XOC3288*	*pilR*	NK3288	III	123.81 ± 20.62	92.96 ± 13.29
*GX_XOC3305*	*colR, vgrR*	NK3305	III	109.52 ± 20.62	84.55 ± 12.94
*GX_XOC3382*	*qseB*	NK3382	III	102.38 ± 20.62	80.85 ± 22.16
*GX_XOC3485*	*gsmR*	NK3485	IV	97.62 ± 16.5	106.56 ± 6.58
*GX_XOC3522*	*–*	NK3522	I	61.9 ± 16.5^*^	101.51 ± 11.13
*GX_XOC3525*	*tctD*	NK3525	II	88.1 ± 20.62	152.42 ± 3.37^*^
*GX_XOC3684*	*hpaR2*	NK3684	II	104.76 ± 16.5	50.89 ± 17.65^*^
*GX_XOC3745*	*ompR family*	NK3745	III	111.9 ± 16.5	107.71 ± 5.92
*GX_XOC3771*	*lytTR family*	NK3771	III	138.1 ± 20.62	83.27 ± 20.97
*GX_XOC3778*	*–*	NK3778	III	130.95 ± 20.62	79.43 ± 14.72
*GX_XOC3779*	*xerR, xibR, xbmR*	NK3779	III	126.19 ± 16.5	153.69 ± 3.25^*^
*GX_XOC3947*	*vieA2*	NK3947	III	123.81 ± 20.62	81.34 ± 14.71
*GX_XOC4109*	*smeR, baeR*	NK4109	II	73.81 ± 8.25^*^	51.6 ± 4.61^*^
		CNK2201	III	89.84 ± 15.6	90 ± 8.18

*^a^Five level is divided according colony morphology of RR mutants on NA with 2% sucrose. ^b^The ratio of colony diameter of each RR mutant to that of wild-type strain, on semi-solid nutrient medium with 0.6% agar. ^c^The ratio of transparent area ratio of each RR mutant to that of wild-type strain, on NA with 1% skim milk. ^∗^ indicates significance at P < 0.05.*

#### Swarming Ability

**FIGURE 2:**
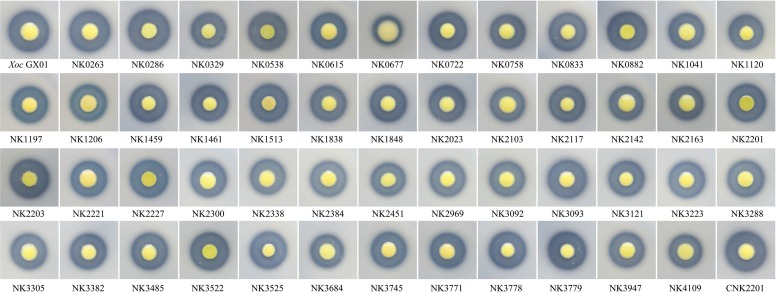
Impact of RR gene mutations on extracellular protease activity of *Xoc*. The strains were inoculated onto NA with 1% skim milk. The difference of extracellular protease could be judged by comparing the availability or size of the hydrolysis transparent ring around the colony, and the results were the average of three independent experiment, listed in **Table 2**.

The results in **Table 2** and **Figure 2** clearly showed that the inactivation of *GX_XOC0538*, *GX_XOC0882*, *GX_XOC1513* (*adeR*), *GX_XOC2201* (*vemR*), *GX_XOC2227* (*ravR*), and *GX_XOC3522* significantly decreased swarming ability, but the mutation in *GX*_*XOC0677* (*sreR*), *GX*_*XOC2142* (*cheY*-*like*), *GX*_*XOC2163* (*cheY*-*like*), *GX_XOC2221*, *GX_XOC2338* (*virG*), *GX_XOC3093* (*pilG*), and *GX_XOC3779* significantly enhanced swarming ability. The results for *vemR* and *ravR* are like the previous studies (He et al., 2009; Tao and He, 2010), but few reports are available for the mutational enhancement in swarming in *Xanthomonas*.

#### Extracellular Protease Activity

**FIGURE 3:**
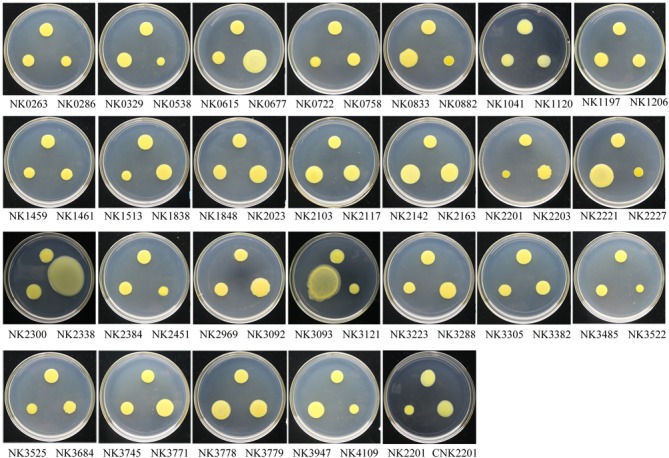
The swarming assays for all RR gene mutants. The culture of each strain was inoculated onto nutrient medium with 0.6% agar in an 18-cm diameter Petri dish, and incubated for 3 days at 28°C. The swarming ability of each strain was measured by colonial diameter and size, and the results were the average of three independent experiment (**Table 2**). The top position in each Petri dish is the colony of wild-type strain, and the name of each mutant was labeled beneath the colony of the corresponding mutant.

The plate assays showed that mutation in RR genes could not totally abolish the extracellular protease activity of the mutants. The difference of extracellular protease had been judged by comparing the availability or size of the hydrolysis transparent ring around the colony on NA containing 1.5% skim milk. The results showed that mutation in *GX_XOC0286*, *GX*_*XOC3525* (*tctD*), and *GX*_*XOC3779* (*xibR*) significantly enhanced protease activity, and mutation in *GX*_*XOC0677* (*sreR*), *GX*_*XOC1041* (*phoB*), *GX*_*XOC2103* (*rpfG*), and *GX_XOC3684* significantly reduced protease activity (**Table 2** and **Figure 3**), of which mutant of *GX*_*XOC0677* (*sreR*) performed the most reduction in protease activity. Compared with the RR mutants in *Xcc* ATCC33913 (Qian et al., 2008b), it was found that more RR genes might be involved in regulation of extracellular protease activity in *Xoc* GX01. These differences may be due to the detailed procedures in method used for assessing the bacterial extracellular protease activity in each assay.

The above results showed that mutation of some RR genes caused multiple phenotypic alterations. Quite a few of RR mutants had the similar tendency in EPS production and swarming ability, either increasing together or reducing together. The mutants of *GX_XOC2103* (*rpfG*) is the only exception, which performed a decreasing in EPS production, but an enhancing in swarming ability. We also found that mutation in *GX_XOC0677* (*sreR*), *GX_XOC2142* (*cheY-like*), and *GX_XOC2221* can increase EPS synthesis or swarming ability, but decrease extracellular enzymes activity. A mutation in *GX_XOC4109 (baeR*) reduced extracellular enzymes activity and swarming ability.

Among the phenotypic altered RR mutants, some genes had been already demonstrated to be involved in either EPS synthesis, extracellular protease activity or swarming of certain *Xanthomonas* strain, while quite a few RR genes might be novel genes that play important roles in regulating the tested virulence determinants of *Xoc*, although the results in this study need further validation. For example, the RR genes without the designation, i.e., *GX_XOC0538*, *GX_XOC0882*, and *GX_XOC3522* should be taken into considerations for their novelty in controlling these virulence determinants.

### Effects of RR Mutations of *Xoc* on Survival Under Environmental Stresses

**FIGURE 4:**
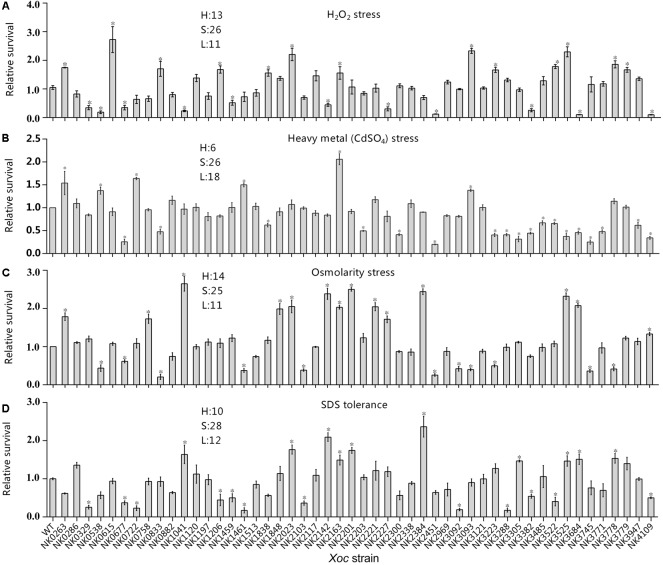
Relative survival of RR gene mutants and wild-type strain under multiple stresses. Stress tolerance of RR mutants was determined using exponentially growing cells. **(A)** Antioxidant reaction (0.125 mM H_2_O_2_); **(B)** heavy metal tolerance (12 μM CdSO_4_); **(C)** osmotic adaptation (300 mM NaCl); and **(D)** sodium dodecyl sulfate (SDS) tolerance (8 × 10^-5^g/ml). Bacterial survival is estimated as the ratio of the number of colonies obtained on NA (3.0 g/L beef extract; 5.0 g/L polypeptone; 1.0 g/L yeast extract; 10.0 g/L sucrose; 8.0 g/L agar pH7.0) with certain antibiotics and incubated for 48 h measured 16 h and none challenge. Relative survival is the ratio of mutant survival to that of the wild-type strain. Standard deviations of three experiments are shown. Significance was tested by Student’s *T*-test (^∗^ indicates significance at *P* < 0.05). Survival of mutants compared with that of wild-type; H, significantly increased; S, similar; and L, significantly decreased.

Stress tolerances of the pathogenic bacteria is important not only for environmental adaptation, but also for the persistent infection, overcoming host immunity responses and niche colonization (Fang et al., 2016). To study the functions of RR genes in stress tolerance, we tested the survival ratios of all mutants obtained under four adverse conditions, including high osmotic pressure, heavy metal stress, and antioxidant reactions and sodium dodecyl sulfate (SDS) lysis. Relative survival of each mutant of RR genes was measured by comparing its survival rates with that of wild type *Xoc* GX01, and the differences significance was tested by Student’s *T*-tests (**Figure 4**).

#### Antioxidant Reaction

Oxidative burst is the major reaction of plants to resistance to the pathogen invasion (Lamb and Dixon, 1997). To infect plants, pathogens have established antioxidant metabolic systems for surviving and spreading in the host plant tissue. In this study, 13 mutants showed higher antioxidant capacity by adding 0.125 mM H_2_O_2_ in the process of bacterial culture and culturing the relative survival of RR mutant after 16 h. In addition, mutations in 11 RR genes have resulted in weakened bacterial antioxidant capacity (**Figure 4A**). It includes the mutant of *GX_XOC2227* (*ravR*, *pdeR*) with GGDEF-EAL domain and two mutants of chemokine-related genes *GX_XOC1459* (*cheB1*) and *GX_XOC2142* (*cheY-like*).

#### Heavy Metal Tolerance

Heavy metals are not only environmental pollutants, but also a kind of germicide commonly used in agriculture. Meanwhile, some plants themselves have developed immune systems dependent on heavy metal ions (Yuan et al., 2010). In this study, all RR gene mutants were inoculated into NB cultures containing 12 μM CdSO_4_ for 16 h culture. The results showed that mutants with six genes, *GX_XOC0263* (*ntrC*), *GX_XOC0538*, *GX_XOC0722*, *GX_XOC1461* (*vieA*), *GX_XOC2163* (*cheY-like*), and *GX_XOC3093* (*pilG*) significantly improved the bacterial resistance to heavy metal stress (**Figure 4B**). In contrast, the resistance to heavy metal tolerance of 18 RR gene mutants is weakened, including *hrpG* mutants NK3223, but the specific mechanisms are not yet known.

#### Osmotic Adaptation

In this study, the osmotic stress was generated by 300 mM NaCl (Zhang et al., 2008). Of all the RR mutants that were constructed, 14 mutants showed a higher ability to tolerance to such osmotic pressure (**Figure 4C**), including *GX_XOC2227* (*ravR*, *pdeR*) and *GX_XOC2201* (*vemR*). Their mutants have been shown to be closely related to bacterial swarming. By contrast, 11 mutants, including *hrpG* and *rpfG* mutants, showed significantly lower survival rates. In addition, mutations in genes such as *GX_XOC2142*, *GX_XOC2163* (*cheY-like*), *GX_XOC2221*, *pilH* and *pilG* associated with bacterial motility and chemotaxis also significantly affect the ability to resist osmotic pressure, indicating the association between osmotic pressure and the regulation of bacterial swimming behavior.

#### SDS Tolerance

Sodium dodecyl sulfate is an anionic surfactant, which can disrupt cell membranes and can instantaneously kill Gram-negative bacteria at high concentration. When SDS concentration was 8 × 10^-5^ g/ml, the relative survival rate of mutants and wild-type strains showed that the SDS stress response of 12 RR gene mutants reduced SDS tolerance (**Figure 4D**), including *rpfG*, which was consistent with previous reports in *Xcc*, while 10 mutants was enhanced in tolerant to SDS, including mutants of *GX_XOC1041* (*phoB*), *GX_XOC2023* (*regA*), *GX_XOC2142*, *GX_XOC2163, GX_XOC2201* (*vemR*), *GX_XOC2384* (*gacA*), *GX_XOC3305* (*colR*, *vgrR*), *GX_XOC3525* (*tctD*), *GX_XOC3684*, and *GX_XOC3778.*

Just like the stress assays of RR mutants in *Xcc* ATCC33913, mutations in some RR genes led to multi-stress tolerance or sensitivity, suggesting that they might play roles to general stress responses. The mutants of *GX_XOC0263* (*ntrC*), *GX_XOC2023* (*regA*), *GX_XOC2163* (*cheY-like*), and *GX_XOC3525* (*tctD*) displayed significantly improved survival rates under at least three kinds of stress conditions, suggesting that they might negatively regulate the general stress responses. Conversely, mutants of *GX_XOC0677* (*sreR*), *GX_XOC2451*, *GX_XOC3382*, and *GX_XOC4109* (*baeR*) showed significantly reduced survival rates under more than three stress conditions, suggesting that they might positively regulate the general stress responses in the bacterium.

In *Xoc* GX01, we also found a four kinds of stresses tolerant mutant, its coding gene *GX_XOC2163* exactly the homologous gene corresponding to *XCC1905*, encoding for a CheY-like, stand-alone REC domain RR. The mutation in *GX_XOC2142*, corresponding to *XCC1886*, also led to a sensitivity to two stresses, i.e., SDS and NaCl, but tolerance to H_2_O_2_, which was not tested in *Xcc*. Interestingly, the mutant of *XCC1187* of *Xcc* ATCC3391, with no homolog in *Xoc* GX01, showed significantly reduced survival under four-stress conditions tested, while the mutant of *GX_XOC4109* (*baeR*) of *Xoc* GX01, with no homolog in *Xcc* ATCC3391, showed significantly altered sensitivity to four-stress conditions.

### Systematic Screening for Virulence-Deficient RR Mutants of *Xoc*

**FIGURE 5:**
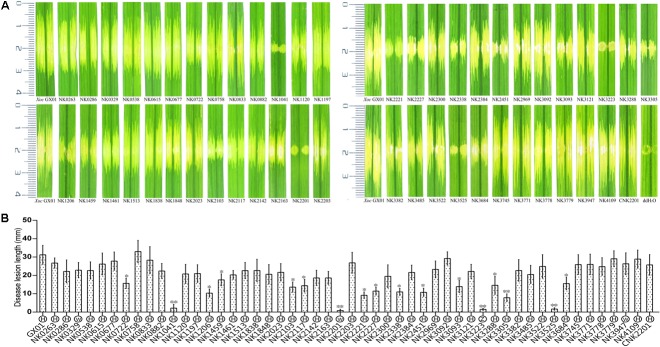
Plant assays of RR gene mutants and other *Xoc* strains. For pathogenicity assays, Rice (*Oryza sativa* L. ssp. *japonica*) cv. Nipponbare was used as host plant. The bacterial suspensions concentration of each *Xoc* strain was adjusted to OD_600_ = 0.5 by the sterilized deionized water. Bacterial suspensions were infiltrated into the rice leaves (6 weeks), on the back of the main vein side, by using needleless syringe. Wild-type strain and sterilized deionized water were used as controls. For hypersensitive response (HR) elicitation test, tobacco (*N. benthamiana*) was used as non-host plant. Significance was tested by Student’s *T*-test (^∗^ and ^∗∗^ indicate significances at *P* < 0.05 and 0.01, respectively). **(A)** Leaf streak disease symptoms caused by *Xoo* strains on inoculated leaves of rice. CNK2201 indicates the complement strain of *GX_XOC2201* mutant. **(B)** The length of disease lesions was measured at 14 days after infiltration inoculation of RR mutants. The results were the average of 20 infiltration spots. The letters in circles beneath the horizontal axis indicate the HR intensities elicited by *Xoc* strains: H, normal HR; W, weak HR; Z, no HR.

To screen RR genes involved in the pathogenicity of *Xoc*, all RR mutants were grown to the exponential stage and the optical density of the cell suspension was diluted to 0.5 at 600 nm with ddH_2_O. The cell suspensions of each mutants were infiltrated into the leaves of the host rice Nipponbare (**Figures 5A,B**). The criteria for identifying virulence reduced mutants is determined by the lesion length of mutant decreased by 40% compared with that of the wild type after difference tests. The results showed that inactivation of 17 RR genes [*cheB1*, *cheB2*, *cheV*, *colR* (*vgrR*), *hpaR2*, *hrpG*, *phoB*, *pilG*, *pilR*, *ravR* (*pdeR*), *rpfG*, *tctD*, *vemR*, *virG*, *GX_XOC0722*, *GX_XOC1206*, and *GX_XOC2451*] significantly reduced the virulence on rice (**Figures 5A,B**), of which, mutants of *hrpG*, *phoB*, *tctD*, and *vemR* almost totally lost the virulence (greater than 90% reduction); mutants of *cheV*, *colR* (*vgrR*), *ravR* (*pdeR*), *virG*, *GX_XOC1206*, and *GX_XOC2451* showed more than 60–80% reduction in virulence than wild type strain; mutants of *cheB1*, *cheB2*, *pilG*, *pilR*, *rpfG*, *GX*_*XOC0722*, and *GX*_*XOC3684* showed more than 40–60% virulence reduction. Of these virulence related RR genes, *cheB* (in *Xoo*, our unpublished data), *colR* (*vgrR*), *hpaR2*, *hrpG*, *ravR* (*pdeR*), *rpfG*, *tctD*, and *vemR* have been demonstrated to be involved in pathogenicity of *Xanthomonas* spp. (Tang et al., 1991; Wengelnik et al., 1996; Qian et al., 2008b ; Zhang et al., 2008; He et al., 2009; Tao and He, 2010; Yang et al., 2012). To make a clear distinction between mutants that have a general growth defect *in vitro*, and mutants that have a specific defect in virulence, we have determined the growth curves of each RR mutants that showed virulence reduced. The results showed that mutants, NK1041 (*phoB*), NK2201 (*vemR*), NK2338 (*virG*), NK3093 (*pilG*), NK3305 (*colR*), and NK3684 (*hpaR2*), have a general growth defect *in vitro*, suggesting that the reduction in virulence of those mutants might be caused by growth defects (**Supplementary Figure S1**). PhoB is activated under low phosphate conditions and phosphate starvation could enhance virulence in various bacterial species (Pratt et al., 2010; Bielecki et al., 2015; Moreira et al., 2015). *virG* is involved in plant signal capturing through the VirA/VirG TCS in *Agrobacterium tumefaciens* (Jin et al., 1993). *cheV*, *pilG*, and *pilR* has been demonstrated to be required for full virulence of some animal bacterial pathogens (Hobbs et al., 1993; Cróinín et al., 2007; Alexander et al., 2010; Francis et al., 2017). *GX_XOC0722*, *GX_XOC1206*, or *GX_XOC2451* are probably novel virulent genes, since their homologous genes have not been found to be involved in virulence in any bacteria.

To detect whether the pathogenicity change was related to T3SS, the ability of all RR mutants to elicit a hypersensitive response (HR) in an intact tobacco (*N. benthamiana*) leaf was tested by using the injection infiltration method (Reimers and Leach, 1991). The results showed that one mutant (insertion in *hrpG*) totally lose the ability to incite HR and 3 mutants (insertions in *vemR*, *GX_XOC0329*, and *GX_XOC3947*) performed weak HR (**Figure 5B**), suggesting these RR genes might be involved in regulation of T3SS system. HrpG is the key regulator of T3SS system in *Xanthomonas* spp., which has already been demonstrated to play crucial roles in bacterial pathogenicity by mainly controlling the expression of *hrp* genes and effector genes (Wengelnik et al., 1996). Inactivation of *hrpG* in any *Xanthomonas* strain tested mostly resulted in a null HR on the appropriate non-host plants and total loss of pathogenicity on host plants. VemR is a standalone RR, containing only a REC domain, which was demonstrated to regulate virulence, exopolysaccharide synthesis and motility in *Xcc* (Tao and He, 2010). Its relatedness with HR elicitation is unknown before. To confirm the relatedness of *vemR* to HR phenotype, we tested HR of the complemented strain of *vemR* mutant on *N*. *benthamiana*. The result showed that the complemented strain could elicit normal HR on the non-host plant (**Supplementary Figure S2**). Given that the disruption of *vemR* gene lead to a sharp decreasing in virulence and a weak HR, we presumed that *vemR* participates in the regulation of the expression of multiple pathogenic factors, including the T3SS/*hrp* system. As for the two unnamed genes, *GX_XOC0329* and *GX_XOC3947*, disruption of each of them lead to a moderate HR and sensitive to stresses, but has no significant effects on full virulence of *Xoc*. *Xoo PXO_02944*, the homolog of *GX_XOC3947*, encodes a negative regulator in virulence, extracellular polysaccharide production and biofilm formation in *Xoo* (Li X.T et al., 2014).

## Discussion

Two-component regulatory system, consisting of HK and RR, is a predominant system in bacteria to sense and adapt to changing environment, and RR is the executor of this signal transduction system (Capra and Laub, 2012). In this study, a genome-scale study of TCS RR genes of *Xoc* GX01 has been carried out by mutational analysis (**Supplementary Table S3**), in which 50 of the 55 annotated RR genes have been mutated using insertion inactivation. From plate morphological assays to plant tests, we have obtained a few RR mutants with significant phenotypic and pathogenicity changes, which unfolded a corner of the complexity of TCS and provided genetic clues for further investigating the biological functions of the TCS genes in adaptation and pathogenesis of this important rice bacterial pathogen.

*Xoc* is a free single cell bacterium that adapted to diverse ecological niches, i.e., soil, water, plant residues, seed surface, and host plants. A better epiphytic growth facilitates its invasion and infection. Constantly exposed to a wide variety of environmental stresses, *Xoc* has developed sophistic TCS systems to senses environmental stimuli and maintains the cellular homeostasis. In this study, we have rated the effects of disruptions of RR genes on environmental responses by examining the viability of RR gene mutants under four kinds of stresses, i.e., oxidative stress, heavy metal stress, osmotic stress, and SDS lysis. Compared with wild type GX01 strain, most of the RR mutants showed significant changes to the stresses, becoming either more tolerant or more sensitive. We found that mutations in different RR genes resulted in similar phenotypes to the certain stress, and inactivation of one RR gene might lead to multiple phenotypical changes. The similar situation has been found in *Xcc* ATCC33913 (Qian et al., 2008b). Some homologous genes in both *Xoc* and *Xcc* performed the similar functions in stress tolerance, for example, *cheB* family, *cheY* family, *gacA*, *pilH*, *regA*, *rpfG*, *sreR*, and *vemR* etc. The mutants of some other homologous genes performed different tolerance, e.g., *ravR* mutant to NaCl, *vgrR* mutant to SDS and *GX_XOC2117* (*XCC1866*) to NaCl. Interestingly, the mutants of strain specific RR genes in *Xoc* or *Xcc* also performed significant phenotypic alterations with that of wild type strain in stress assays. Especially, the mutant of *XCC1187* of *Xcc* ATCC3391 (Qian et al., 2008b), with no homolog in *Xoc* GX01, showed significantly reduced survival under four-stress conditions tested, while the mutant of *GX_XOC4109* (*baeR*) of *Xoc* GX01, with no homolog in *Xcc* ATCC3391, showed significantly altered sensitivity to four-stress conditions. These findings just shed a glimpse into the complexity, multiplicity and function diversity of TCS in stress adaptation of *Xanthomonas*, further indicating that a bacterium can react simultaneously to many kinds of stresses and the various stress response systems interact in global regulatory networks (Fang et al., 2016).

The genomic analysis showed that *Xoc* strains possess a serial of pathogenicity and virulence factor genes encoding for the secretion systems, type III secretion effectors, exocellular enzymes, exopolysaccharide, lipopolysaccharide synthesis and adhesins. During the interactions between pathogens and host plants, the regulator proteins do not need to directly act on host cells, however, they play important roles in regulation of the virulence genes at certain spatial and temporal conditions (Büttner and Bonas, 2010). In this study, three kinds of virulence factors, i.e., extracellular polysaccharides (EPS), extracellular protease, and swarming ability, were selected as examples to assess the involvement of RR genes in regulation of the virulence genes. Like the results of stress assays, the mutations of some RR genes caused multiple phenotypic alterations. Quite a few of RR mutants had the similar tendency in EPS production and swarming ability, either increasing together or reducing together. The mutants of *GX_XOC2103* (*rpfG*) is the only exception, which performed a decreasing in EPS production, but an enhancing in swarming ability. We also found that mutation in *GX*_*XOC0677* (*sreR*), *GX*_*XOC2142* (*cheY*-like) and *GX*_*XOC2221* can increase EPS synthesis or swarming ability, but decrease extracellular enzymes activity. A mutation in *GX*_*XOC4109* (*baeR*) reduced extracellular enzymes activity and swarming ability, which is absent from *Xcc* ATCC33913. Among the phenotypic altered RR gene mutants, some genes had been already demonstrated to be involved in either EPS synthesis, extracellular protease activity or swarming of certain *Xanthomonas* strain, while quite a few RR genes might be novel genes that play important roles in regulating the above virulence determinants of *Xoc*, although the results in this study need further validation, for example, the RR genes unnamed, i.e., *GX_XOC0538*, *GX_XOC0882*, and *GX_XOC3522*.

Both *Xoc* and *Xcc* are very important bacterial phytopathogens. Comparatively, *Xcc* ATCC33913 and *Xoc* GX01 possess same amount of TCS genes, 54 RR genes in each genome, 46 shared by them. In this study, we found that about one-third of RR genes, 17 RR genes, might be involved in virulence of *Xoc*, while in *Xcc* ATCC33913, only four RR genes have been identified as virulence genes (Qian et al., 2008b). In total, 31.5% of TCS genes in *Xoc* GX01 were responsible directly or indirectly for pathogenesis, however, this ratio is only 7.4% in *Xcc*. The reason for such distinctions between *Xcc* and *Xoc* might be the differences in hosts, bacteria, inoculation methods, and the statistical methods, and functional redundancy between certain genes (Alfano and Collmer, 1996). The extent of the involvement of TCSs in controlling virulence is unclear for most of pathogenic bacteria. It is estimated that over 50% of TCSs implicate in controlling either virulence or virulence-related behaviors of *Pseudomonas aeruginosa* (Francis et al., 2017). Genome-scale dissecting the relatedness of TCSs and bacterial virulence will facilitate to reveal the mechanisms of multi-regulation networks of TCSs in controlling bacterial adaptation and virulence.

In *Xanthomonas*, 12 RR genes have been identified to be related to bacterial pathogenesis, including *cheB* (our unpublished data in *Xoo*), *colR* (*vgrR*) (Qian et al., 2008b; Zhang et al., 2008), *gsmR* (Liu et al., 2013), *hpaR2* (Li R.F et al., 2014), *hrpG* (Wengelnik et al., 1996), *pcrR* (Wang et al., 2017), *phoP* (Lee et al., 2008), *ravR* (*pdeR*) (He et al., 2009; Yang et al., 2012), *raxR* (Burdman et al., 2004), *rpfG* (Tang et al., 1991), *vemR* (Tao and He, 2010), and *xerR* (also named *xibR* and *xmbR*) (Wang et al., 2011; Yaryura et al., 2015; Pandey et al., 2016). RRs can modulate the expression of various virulence factors by binding DNA, combining protein and participating in the c-di-GMP metabolism, generating a high level of regulatory diversity. In this study, we also found that mutations in *cheB1*, *cheB2*, *colR* (*vgrR*), *hpaR2*, *hrpG*, *ravR* (*pdeR*), *rpfG*, and *vemR* of *Xoc* significantly reduced the virulence on rice. However, mutations in *gsmR*, *pcrR*, *raxR*, and *xibR* did not affect the virulence of *Xoc* on rice, implying that these genes may have a different function in *Xoc*. A similar situation happened in the study of TCSs of *Xcc* ATCC33913. Mutations in the ortholog of virulence-associated *raxR* of *Xoo* or *gacA* of *P. aeruginosa* (Parkins et al., 2001; Burdman et al., 2004) in *Xcc*, did not affect the bacterial virulence. Even the HrpG, the key regulator of T3SS/*hrp* system in *Xanthomonas*, an insertion in *hrpG* only has limited effects on the virulence of *Xcc* ATCC 33913, which might be associated with specific bacterial strains, or the residual activity in the *hrpG* mutant obtained by using insertional mutagenesis (Qian et al., 2008b). Since the functional analysis of *cheB*, *colR*, *hpaR2*, *hrpG*, *pdeR*, *ravR*, *rpfG*, and *vemR* have been intensively reviewed (Qian et al., 2008a,b; Wang et al., 2010; Li R.F et al., 2014), we will focus on the probably novel virulence related RR genes of *Xanthomonas* in subsequent discussion, including *phoB*, *pilG*, *pilR*, *tctD*, *virG*, *GX*_*XOC0722*, *GX*_*XOC1206*, and *GX*_*XOC2451*.

*phoB* encodes an OmpR family RR contains a REC domain with a transcriptional regulatory C-terminal and regulates virulence in various bacterial species (Bielecki et al., 2015). PhoB is activated under low phosphate medium conditions. PhoB-mediated gene activation or repression depends on PhoB phosphorylation and its binding to Pho-boxes, the promoters of genes encoding mainly phosphate starvation system (Pst) (Chekabab et al., 2014). Although there is no direct experimental evidence linking *phoB* to *Xanthomonas* virulence, functional genomics study showed that PhoB-PhoR regulates a network connecting phosphate homeostasis and virulence expressions in *Xac* (Pegos et al., 2014; Moreira et al., 2015, 2017). *tctD* also encodes an OmpR family RR contains a REC domain with a transcriptional regulatory C-terminal and was found to positively regulate the expression of *citH* that is specifically needed for *Xcv* to accumulate citrate and is required for full virulence of *Xcv*. However, the *tct* mutant of *Xcv* did not show virulence reduction compared with the wild type strain, but affected the ability of *Xcv* to grow on citrate (Tamir-Ariel et al., 2011). Cross talks between PhoB and TctD were found in *P. aeruginosa*. The two RRs were phosphorylated by distinct histidine that sense the availability of phosphate and carbon source, respectively, indicating that the bacterial pathogen may use cross-regulation to adapt its behavior to complex niches (Bielecki et al., 2015). Interestingly, in this present study, inactivation either *phoB* or *tctD* resulted in significantly reduced in virulence, which needs further confirmation. *pilG* encodes CheY family RR containing a REC domain, and *pilR* encodes a NtrC family RR containing a REC domain, a Sigma-54 interaction domain and a DNA-binding domain at C-terminus. PilG activates the adenylate cyclase (CyaB) and the pilus extension ATPase, regulating pilus extension. PilR regulates transcription of the major pilin genes (Francis et al., 2017). In this study, insertions in *pilG* or *pilR* led to a moderate virulence decreasing. However, the mutants performed significantly enhanced swarming ability, which differs from the results in *P. aeruginosa*, of which the *pilR* mutant was unable to swarm (Köhler et al., 2000). *Xoc* can produce a large amount of EPS which facilities the flagella-based motility. We speculated that insertion in *pilG* or *pilR* reduced twitching by inhibiting the type IV pili, but conversely enhanced swarming ability of the mutants. *virG* encodes an OmpR family RR containing an REC and a C-terminal DNA-binding region. VirG is involved in plant signal capturing through the VirA/VirG TCS in *A. tumefaciens* (Jin et al., 1993). *virG* mutant showed significantly reduced in virulence, but greatly increased in EPS production and swarming ability (**Figure 3**). Amazingly, the mutants increased in stress tolerance, EPS production, swarming or protease activity did not always show enhanced in virulence, conversely, some of which performed significantly decreased in virulence, for example, mutants of *phoB*, *vemR*, and *tctD*, in addition to *virG*. Bacterial optimal virulence is the combination result under long-term evolutionary selections. It can be speculated that any abnormal changes in physiology could lead to defective in full virulence of a bacterial pathogen. We also noticed that not all the mutants of *Xoc* performing significant reduction in stress tolerance or reduction in virulence determinants always showed significantly decreasing in virulence on rice, for example, mutants of *baeR*, *sreR*, and *GX_XOC0538.* Especially, the mutant of *sreR* performed a relatively severe (greater than 50%) reduction in protease activity, however, its virulence on rice was not affected. The possibility is that the infiltration inoculation directly introduced the bacterial suspension into the intercellular spaces of the mesophyll of rice leaves, where the bacteria could easily colonize the niche. We have already found that some of this kind of mutants showed significant reduction in infection rate on rice by spraying inoculation. These are the questions we will be addressing in our ongoing studies.

Of the three probably novel virulence related RR genes, *GX_XOC0722* encodes an OmpR family RR containing a REC and a C-terminal DNA-binding region; *GX_XOC1206* encodes a NarL family RR containing a REC and an HTH motif at the C-terminal DNA-binding region; *GX_XOC2451* encodes an unclassified RR contains a single REC. In addition to virulence reduction, inactivation of each of them resulted in a significant alteration in stress tolerance or in production of virulence factors, but not in eliciting HR. The mechanistic basis of these genes required for bacterial full virulence remains to be determined. However, prerequisitely, the RR genes identified require confirmation by genetic restoration, since the reduced-virulence phenotypes may be due to spontaneous ectopic mutations.

In the present study, we genetically mutated 50 of the total of 54 RR genes of *Xoc* GX01 using insertion inactivation, and identified a few candidates of virulence related RR genes by screening phenotypic changes and plant assays. Rice bacterial leaf streak is a growing and expanding destructive rice disease, which can serve as a representative pathosystem for revealing non-vascular pathogenesis in other plants (Wang et al., 2007). The screening for candidates of virulence related RR genes of *Xoc* is the first step toward revealing the mechanisms of the complex, overlapped and crossly linked TCSs in the regulation of bacterial pathogenesis, especially in the early stage of infection. Although the results in this study need further verification, the results provided experimental clues for further investigating the biological functions of TCS genes in *Xoc*, serving as a starting point for reconstructing the signaling networks in the adaptation and pathogenesis of this important bacterial pathogen.

## Author Contributions

Y-QH, J-LT, and Z-CZ conceived and designed the study. Z-CZ, MZ, L-DX, X-NN, H-PQ, M-LL, and G-HH constructed the mutants. H-PQ constructed the complemented strain. Z-CZ, MZ, L-DX, and G-HH conducted the virulence tests. Z-CZ, X-NN, H-PQ, M-LL, and L-DX performed the phenotypic and stress assays. Y-ML and WJ performed the bioinformatic analyses. Z-WJ and XY carried out the growth curve assays of the RR mutants. Y-QH, J-LT, and Z-CZ analyzed the data. Y-QH and Z-CZ wrote the manuscript. J-LT revised the manuscript. All authors read and approved the final manuscript.

## Conflict of Interest Statement

The authors declare that the research was conducted in the absence of any commercial or financial relationships that could be construed as a potential conflict of interest. The handling editor and reviewer H-LW declared their involvement as co-editors in the Research Topic and confirm the absence of any other collaboration.

## Footnotes

1 www.p2cs.org

## Supplementary Material

FIGURE S1 Growth curves of 17 RR mutants in NB medium. 17 RR mutants and wild-type strain cultured in NB, respectively. Bacterial density was measured by the absorbance at OD_600_, and shown as the mean of three experimental reduplicates ± the standard deviation. **(A)** Growth curves of NK0722, NK1041, NK1206, NK1459, and GX01. **(B)** Growth curves of NK2103, NK2117, NK2201, NK2221, and GX01. **(C)** Growth curves of NK2227, NK2338, NK2451, NK3093, and GX01. **(D)** Growth curves of NK3223, NK3288, NK3305, NK3525, NK3684, and GX01.

FIGURE S2 The HR tests of *vemR* mutant and its complemented strain. For hypersensitive response (HR) elicitation test, tobacco (*Nicotiana benthamiana*) was used as non-host plant. The name of each *Xoc* strain was marked beneath the infiltrated area.

TABLE S1 Two component regulatory system genes distributed in curtain *Xanthomonas* ssp.

TABLE S2 Primers used in this study.

TABLE S3 The phenotypes of RR mutants of *Xoc* GX01.
