# Supplementary figures and images for: Genome-Wide Screening for Novel Candidate Virulence Related Response Regulator Genes in Xanthomonas oryzae pv. oryzicola
Source: Front Microbiol. 2018 Aug 7;9:1789. doi: 10.3389/fmicb.2018.01789 (PMC6090019; doi:10.3389/fmicb.2018.01789)

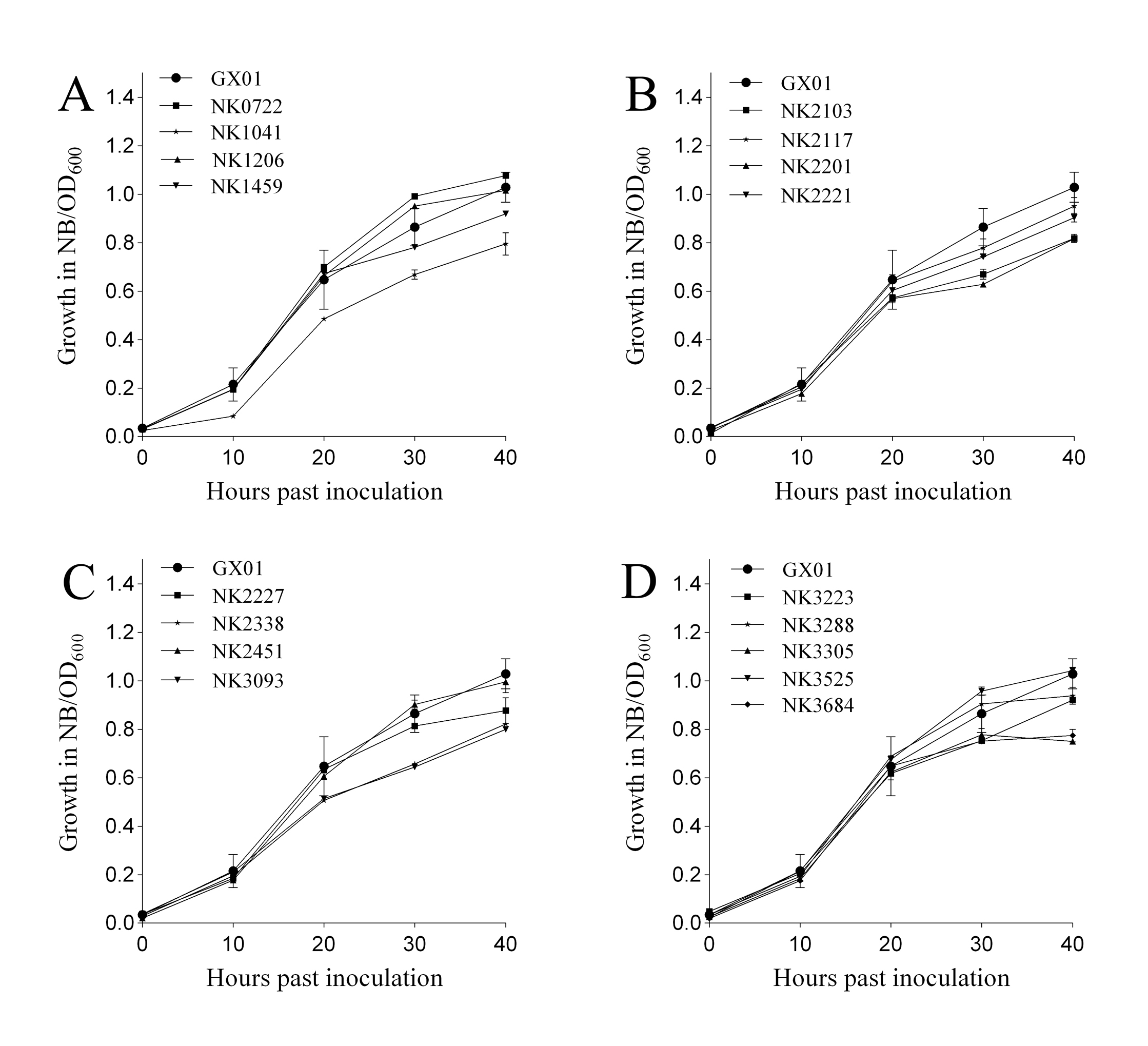

Supplement: FIGURE S1 — Growth curves of 17 RR mutants in NB medium. 17 RR mutants and wild-type strain cultured in NB, respectively. Bacterial density was measured by the absorbance at OD600, and shown as the mean of three experimental reduplicates ± the standard deviation. (A) Growth curves of NK0722, NK1041, NK1206, NK1459, and GX01. (B) Growth curves of NK2103, NK2117, NK2201, NK2221, and GX01. (C) Growth curves of NK2227, NK2338, NK2451, NK3093, and GX01. (D) Growth curves of NK3223, NK3288, NK3305, NK3525, NK3684, and GX01. [file Image_1.JPEG]

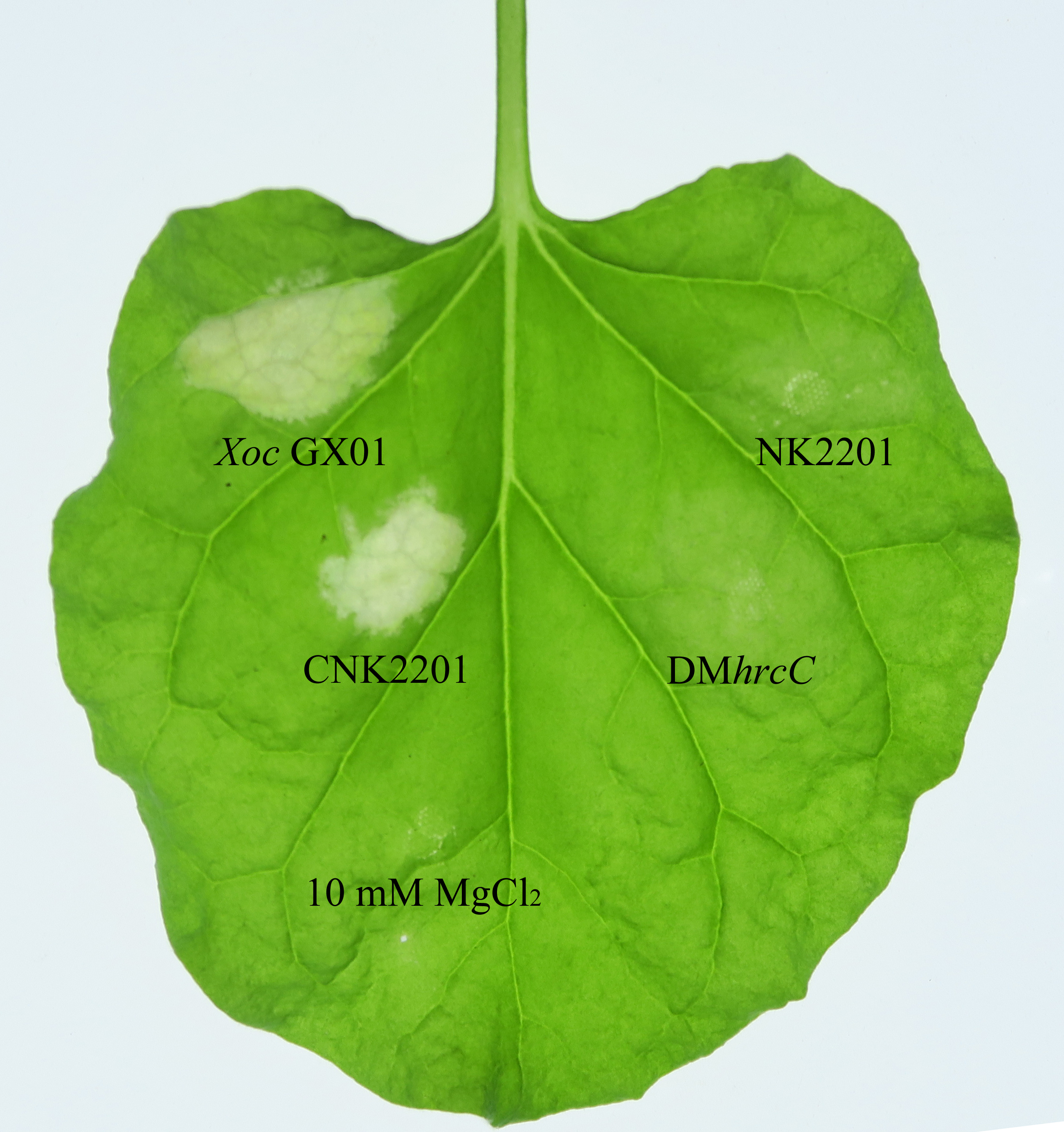

Supplement: FIGURE S2 — The HR tests of vemR mutant and its complemented strain. For hypersensitive response (HR) elicitation test, tobacco (Nicotiana benthamiana) was used as non-host plant. The name of each Xoc strain was marked beneath the infiltrated area. [file Image_2.JPEG]
