# Supplementary material for: Genome-Wide Screening for Novel Candidate Virulence Related Response Regulator Genes in Xanthomonas oryzae pv. oryzicola
Source: Front Microbiol. 2018 Aug 7;9:1789. doi: 10.3389/fmicb.2018.01789 (PMC6090019; doi:10.3389/fmicb.2018.01789)
Supplement: TABLE S2 — Primers used in this study. [file Table_2.DOCX]

**TABLE S2.** **Primers used in this study**

| Primers | Sequence (5'-3') | **For construction** |
| --- | --- | --- |
| F_0263 | CCCAAGCTTCCTGACCCTGCTCGACAAGC | NK0263 |
| R_0263 | CCCAAGCTTAGGCACCGGTTTCGTGGC |  |
| F_0286 | CCCAAGCTTGTGCATCCTGATCATCGA | NK0286 |
| R_0286 | CCCAAGCTTAGTTGACCGCAACGAACG |  |
| F_0329 | CCCGAATTCATGACCATCCCTTGTTTCG | NK0329 |
| R_0329 | CCCAAGCTTTCGCGTTCTTCGCTGTC |  |
| F_0538 | CCCAAGCTTAGCAGCATCTGGTGGAGC | NK0538 |
| R_0538 | CCCAAGCTTACCTTGGTCACCAGCAGG |  |
| F_0615 | CCCAAGCTTGCTGGATTACCTGATGAAACC | NK0615 |
| R_0615 | CCCAAGCTTTAATTGATCGCGCAGGCT |  |
| F_0677 | CGCGGATCCCGCCATCCGTACCATTCTT | NK0677 |
| R_0677 | CCCAAGCTTCACCTGCTTGAGCACTTCCAG |  |
| F_0722 | CCCAAGCTTACGACGACCCTGATCTGC | NK0722 |
| R_0722 | CCCAAGCTTGGGTGTTCGACAAAGGTG |  |
| F_0758 | GGGTCTAGAGACTGACCTCACTGCCATCC | NK0758 |
| R_0758 | GGGAAGCTTGAACACGCTTTCTTCCACC |  |
| F_0833 | CGCGGATCCAATGATCGGCGAATACCTGG | NK0833 |
| R_0833 | CCCAAGCTTAGCTCCTGAATCGCAAACG |  |
| F_0882 | CCCAAGCTTTGATTGCCACCACCGATCC | NK0882 |
| R_0882 | CCCAAGCTTGCGCTTGAACAGTACGGT |  |
| F_1041 | CCCAAGCTTGATCGTCGATGACGAACC | NK1041 |
| R_1041 | CCCAAGCTTAGCAGGCGATATTCGGTC |  |
| F_1120 | CGCGGATCCACGAAATCATCATCGCCACC | NK1120 |
| R_1120 | CCCAAGCTTCCATCACTTCCAGCAGTTCATCTT |  |
| F_1197 | CCCAAGCTTGCTGGTCATTGAAGACAACC | NK1197 |
| R_1197 | CCCAAGCTTGATACAGATGCAGCACCT |  |
| F_1206 | CCCAAGCTTAGATTCCGCAGATCCAGC | NK1206 |
| R_1206 | CCCAAGCTTGTTCTTGATGGTGCCCTC |  |
| F_1459 | CGCGGATCCTCGTGCTGGATGTGGAAAT | NK1459 |
| R_1459 | CCCAAGCTTACTGCAATCTGGCACAAGC |  |
| F_1461 | CCCATCGCTGTTGCTGGTGGA | NK1461 |
| R_1461 | CCCAAGCTTTCGGTGAAGGGAATGCTG |  |
| F_1513 | CCCAAGCTTTTCTGCTGGTAGAAGACGACG | NK1513 |
| R_1513 | CCCAAGCTTGTCGGTGTCAAATCCAGC |  |
| F_1838 | CCCGGATCCGCTGGTGTTCGCCAAATC | NK1838 |
| R_1838 | CCCAAGCTTAATGCCTCACGGAAATGC |  |
| F_1848 | CCCGGATCCGTGGTGGTCAGTGGCGTGTA | NK1848 |
| R_1848 | CCCAAGCTTTAAGCCGGTGAGGAAGTCG |  |
| F_2023 | CCCAAGCTTTCGACGACGACACCTTGTA | NK2023 |
| R_2023 | CCCAAGCTTATATGCTCCCACTGCAGG |  |
| F_2103 | CCCGGATCCTGTTGCTGCTGGACTACCG | NK2103 |
| R_2103 | CCCAAGCTTGCGTTTCATCACGCTCATCT |  |
| F_2117 | CCCGGATCCGCTGGAAGGTTATGCGGAAGA | NK2117 |
| R_2117 | CCCAAGCTTGCATTTGCGGCGACAGA |  |
| F_2142 | CGCGGATCCGATGGTGTCTTTCGCCCTTAC | NK2142 |
| R_2142 | CCCAAGCTTTTTGCCTTCGGACTTCTTGT |  |
| F_2163 | CGCGGATCCCCTGATCGTGGACGACTTCT | NK2163 |
| R_2163 | CCCAAGCTTGGTGAACGGCTTGATGATGT |  |
| F_2201 | CGCGGATCCGCTCAATATCGTGGGCAGTG | NK2201 |
| R_2201 | CCCAAGCTTTGCTCGGCATCATCGAAGTG |  |
| F_2203 | CCCAAGCTTCCATGACCGACGTACTGC | NK2203 |
| R_2203 | CCCAAGCTTAGCAGCACCAGATCGTTG |  |
| F_2221 | CCCTCTAGAGGCCCAGGTTATCTGGTTG | NK2221 |
| R_2221 | CCCAAGCTTATTTCCGTCGTCAGCGTAT |  |
| F_2227 | CCCGGATCCATTGCCGCTGTCGGTGTT | NK2227 |
| R_2227 | CCCAAGCTTTCGTGGACGAAGTTGACGT |  |
| F_2300 | CGCGGATCCTCGACGACGATGCTTCGAT | NK2300 |
| R_2300 | CCCAAGCTTCGTTCTTCATCGCTTCCAC |  |
| F_2338 | TGCTCTAGACAAGCTGCATTCCCGTTCCT | NK2338 |
| R_2338 | CCCAAGCTTAATGGCTTGGGCAGGTAGTC |  |
| F_2384 | CGCGGATCCCGTGCGTACAGGCATGAAGA | NK2384 |
| R_2384 | CCCAAGCTTGCACGGAGACAATGATGACG |  |
| F_2451 | CCCAAGCTTATGCCATGCTGCTCGACCTGCA | NK2451 |
| R_2451 | CCCAAGCTTCCCTGGACAACGCGCCAA |  |
| F_2969 | CGCGGATCCATCCCATCCACGTCTATTGC | NK2969 |
| R_2969 | CCCAAGCTTTCGGAGGTCAGCGGTTTGAT |  |
| F_3092 | CGCGGATCCCAGCGCAGTCTTCAGTCAAT | NK3092 |
| R_3092 | CCCAAGCTTATGTAGTCGCTGGCACCTTG |  |
| F_3093 | CGCGGATCCACTCGCAGGACTGAAGGTG | NK3093 |
| R_3093 | CCCAAGCTTAAGAGCTGGTTGCCCTTG |  |
| F_3121 | CGCGGATCCGAGACCTGCTCATGCCAC | NK3121 |
| R_3121 | CCCAAGCTTGGGCAGGTTATACGGTGCTT |  |
| F_3223 | CGCGGATCCCTCGGTGTTTTCCGATGAAC | NK3223 |
| R_3223 | CCCAAGCTTGCAACTGGTATGGACCAACG |  |
| F_3288 | CCCAAGCTTTCGATTTCGTCAGCAAACC | NK3288 |
| R_3288 | CCCAAGCTTTTCGTCCAGGAACAAGGT |  |
| F_3305 | CCCAAGCTTGTCACACGGTGGACTTCG | NK3305 |
| R_3305 | CCCAAGCTTCCAGCGTGTCCAGGTTGT |  |
| F_3382 | CGCGGATCCGCATTCTGCTGGTTGAAGACG | NK3382 |
| R_3382 | CCCAAGCTTATTGGTGTCGAACGGCTTG |  |
| F_3485 | CGCGGATCCCTTGCGTATTCTGTTCGTTGG | NK3485 |
| R_3485 | CCCAAGCTTAGCGCAATACGAATGGTGTCC |  |
| F_3522 | CGCGGATCCCATGGCCGATCTCACTATTCT | NK3522 |
| R_3522 | CCCAAGCTTCGGATGATCGTTGGAGGAG |  |
| F_3525 | CCCAAGCTTTGCTGGTCGAAGACAACG | NK3525 |
| R_3525 | CCCAAGCTTTCCAGCGTCAAGGTATGC |  |
| F_3684 | CCCAAGCTTCAGCTCCGTCAGCGTCAC | NK3684 |
| R_3684 | CCCAAGCTTCCGCTTCGAACTTGCCTG |  |
| F_3745 | CGCGGATCCTGTCTATCCGTGCTTCCCTAC | NK3745 |
| R_3745 | CCCAAGCTTTCAACACGACGATGCCCAGT |  |
| F_3771 | CGCGGATCCTGGTGTTCGTCACTGCCTAC | NK3771 |
| R_3771 | CCCAAGCTTTCCAGCACATCCAGCAGTTC |  |
| F_3778 | CGCGGATCCTCTTGTGGTCGAGGACGATT | NK3778 |
| R_3778 | CCCAAGCTTGTTGTAGGGCTTCATGCAGTAG |  |
| F_3779 | CGCGGATCCGGAGTTCTGCGATCAGGTGGTT | NK3779 |
| R_3779 | CCCAAGCTTTCACGATGCTGTCGCTTTTGC |  |
| F_3947 | CGCGGATCCGAGATCCGGTTGTTTCCAC | NK3947 |
| R_3947 | CCCAAGCTTGATCCGGGTGATAGTCCTG |  |
| F_4109 | CCCAAGCTTACGACTGGATCGCCGATG | NK4109 |
| R_4109 | CCCAAGCTTCAGCAGGCAGAACGCAAC |  |
| CF_2201 | CGCGGATCCTCGATGCGCTACGAGACCCT | CNK2201 |
| CR_2201 | CCCAAGCTTTGAAGTCCATAAATTCGAGCAG |  |
| F_pK18mobCOM | GCCGATTCATTAATGCAGCTGGCAC | Mutant confirmation |
